# People with newly diagnosed multiple sclerosis benefit from a complex preventative intervention—a single group prospective study with follow up

**DOI:** 10.3389/fneur.2024.1373401

**Published:** 2024-04-10

**Authors:** Natália Hrušková, Kateřina Berchová Bímová, Angela Davies Smith, Tereza Škodová, Marie Bičíková, Lucie Kolátorová, Ivana Štětkářová, Ľuba Brožek, Alena Javůrková, Gabriela Angelová, Kamila Řasová

**Affiliations:** ^1^Department of Rehabilitation Medicine, Third Faculty of Medicine, Charles University, Prague, Czechia; ^2^Department of Applied Ecology, Faculty of Environmental Sciences, Czech University of Life Sciences, Prague, Czechia; ^3^MS Research, Treatment and Education, The Vassall Centre, Bristol, United Kingdom; ^4^Department of Steroids and Proteofactors, Institute of Endocrinology, Prague, Czechia; ^5^Department of Neurology, Third Faculty of Medicine, Charles University, University Hospital Kralovske Vinohrady, Prague, Czechia; ^6^Mediterra s.r.o., Malvazinky Rehabilitation Clinic, Prague, Czechia

**Keywords:** multiple sclerosis, fatigue, satisfaction with life, cognitive behavioural therapy, physical therapy, aerobic training, neuroactive steroids

## Abstract

**Background:**

Newly diagnosed people with multiple sclerosis frequently report fatigue, pain, depression and anxiety. Preventative programmes may be beneficial, but there is limited evidence of their effectiveness, especially long-term follow-up.

**Methods:**

The programme consisted of 6-month face to face intervention (an introductory workshop, psychology-led group sessions and individual physical therapy) followed by 6-month self-guided therapy. Outcome measures were taken at baseline, 6 and 12 months. Primary outcomes measures were self-report questionnaires for fatigue, satisfaction with life and disease acceptance. Secondary outcomes were spirometry, spiroergometric parameters and neuroactive steroid levels.

**Results:**

From 22 participants enrolled, 17 completed the first 6 months and 13 the follow-up. Fatigue measured on the Fatigue scale for motor and cognitive functions decreased significantly at 6 months (*p* = 0.035) and at follow-up (*p* = 0.007). The Modified Fatigue Impact Scale (*p* = 0.035) and Satisfaction With Life Scale (*p* = 0.007) significantly increased at follow-up. Spirometry, spiroergometric parameters, steroid hormones and neuroactive steroids levels did not change significantly.

**Conclusion:**

This programme reduces fatigue and improves satisfaction with life in this patient group with improvements sustained at 12 months. People who participated more frequently showed greater benefit.

**Clinical rehabilitation impact:**

The paper describes the effects of a complex preventative intervention for people with newly diagnosed Multiple Sclerosis. The study found that this programme reduces fatigue and improves satisfaction with life with long-term benefit (at 12-month follow up). The individuals who participated less frequently experienced fewer benefits.

## 1 Introduction

Pain, fatigue, depression and anxiety are common symptoms in people newly diagnosed with multiple sclerosis (pwMS) (1–5). This can limit family, social, recreational and work-related activities and negatively influence quality of life (6, 7).

Preventative programmes for newly diagnosed pwMS are frequently recommended (4, 8, 9) and their effectiveness have been documented by several studies (10–16). Some emphasise the need for appropriate information giving (10–12), others utilise different psychotherapeutic interventions such as cognitive behavioural strategies (13) or self-management (14) as well as online psychological interventions (15, 16). Unfortunately, the limited number of studies and the heterogeneity of health outcomes cause difficulties in making clear conclusions for daily practise.

This paper presents a complex preventative programme for early MS (COPREMS) that was developed to attempt to address these issues in newly diagnosed pwMS, which includes a group-based cognitive-behavioural intervention (10, 13) to promote quality of life and psychological well-being, an information workshop (13), and also a physiotherapy programme, which is innovative. Suitable physical activity and targeted physiotherapy aimed at slowing down the progression of the disease, restoring function, maintaining self-sufficiency and improving the quality of life (17) were included. This comprised of three components: (1) Aerobic training to reduce deconditioning which is known to increase fatigue in MS (12) and to improve physical fitness and ability to execute activities of daily living (13, 14). (2) Treatment of any musculoskeletal issues which can contribute to MS related pain (1), for example back pain is described in 41.6% pwMS and is present in the early stages of the disease (15). (3) Neuroproprioceptive facilitation and inhibition techniques which aim to enhance the effectiveness of synaptic connections between neurones forming functional networks (15). The study was conducted over 6 months as long-term programmes for newly diagnosed pwMS are recommended (1, 2, 13, 18, 19) with a further follow-up at 1 year.

The aim of this study was to evaluate the effectiveness of COPREMS, both immediately following the intervention and in the longer term. It was important to capture the perceptions of the participants regarding the impact of the intervention and so the following were used as primary outcome measures, Fatigue Scale for motor and cognitive functions, Modified Fatigue Impact Scale, Beck Depression Inventory, Satisfaction with Life Scale and Multiple Sclerosis Acceptance Questionnaire. Secondary outcomes were spiroergometric parameters and neuroactive steroid levels, chosen based on authors’ previous experience (17, 20, 21). Spiroergometric parameters give information about physical condition and neuroactive steroids can be considered indicators of disease progression because of their important role in the pathogenesis of MS as they reduce inflammatory processes, modulate cellular immunity, have neuroprotective effects, play a role in myelination and in the priming of synaptic plasticity induction. In conjunction with other hormones and transmitters, they affect some aspects of human mood, emotions and behaviour, and as such increase feelings of well-being (17).

## 2 Materials and methods

This study was approved by the ethical commission for research prior to commencement (Kralovske Vinohrady University Hospital Ethics Committee, Prague EK-VP/25/0/2014).

### 2.1 Design

A single group prospective study with follow-up (NCT04667130). People recently diagnosed with MS undertook a 6-month preventative comprehensive programme, followed by 6-month self-guided therapy. Outcome measures were taken at the start, at 6 months and at 1 year follow-up.

### 2.2 Participants

Participants were recruited from October 2017 to October 2019 by neurologists at neurology departments in Prague according to the inclusion criteria: adults (18–60 years old) with definite MS, newly diagnosed (0.5–3.5 years since diagnosis), stable clinical condition and with stable treatment over the previous 3 months (Table 1). Patients with additional pathology were excluded. All participants gave written consent.

**Table 1:** Baseline characteristics.

Characteristics	
Age (years)	37.6 ± 10.3
Sex: female/male	14 (82%)/3 (18%)
BMI	24.74 ± 4.10
EDSS	2.41 ± 0.9
Time since onset (years)	2 ± 1.1
Type of MS:	RR 17(100%)
Pharmacotherapy: DMD/corticosteroids/none	13 (76%)/3 (18%)/1 (6%)

BMI, Body mass index; EDSS, Expanded disability status scale; MS, Multiple sclerosis; DMD, Disease modifying drugs.

### 2.3 Intervention

**Figure 1:**
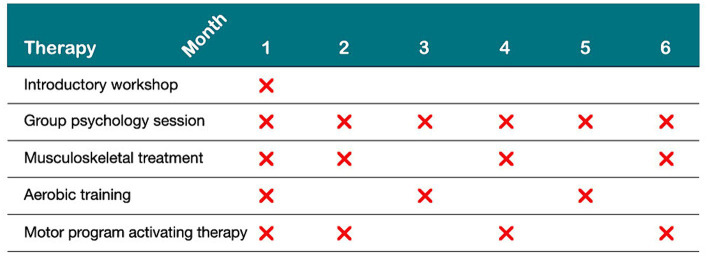
Distribution of interventions during the first 6 months.

Six months face to face treatment comprising one 2-h introductory workshop, six 2-h group psychotherapy sessions and 10 1-h individual physical therapy sessions. This was followed by 6 months of self-guided therapy. Participants were invited to a total of 18 sessions (Figure 1) and were encouraged to apply learned strategies to everyday life. Therapists were flexible with therapy dates to maximise attendance and maintained contact with participants to give timely reminders so that participants would complete all offered therapy sessions.

Sessions were led by an experienced psychotherapist and physical therapists (minimum 2 years’ practise with pwMS) specially trained in each intervention. Treatments were of moderate intensity and individualised. Therapy took place at the Department of Neurology, Kralovske Vinohrady University Hospital in Prague.

At the introductory 2-h workshop, the participants were introduced to the entire programme in detail using a PowerPoint presentation. The therapists responsible for each intervention presented the scientific evidence and explained the potential therapeutic benefit. The study goals were to improve participants’ acceptance and adjustment to MS, to take an active approach to help stabilise the disease course, and to gain understanding of the need to seek timely treatment to offset the development and impact of future symptoms. Participants received a booklet explaining the programme in detail including photographs of the therapy elements. This was to aid understanding and to better enable participants to practise learned techniques between sessions and help them to become embedded in daily life.

Group psychotherapy sessions took place monthly using cognitive-behavioural techniques. Each session started with relaxation followed by psychotherapy which focused on: (a) the experience of MS diagnosis (how the participants felt when receiving the diagnosis and how they feel at present), identity change and redefinition following the diagnosis (in the family, work and free-time domains); (b) life goals to give participants a sense of coherence (before and after the diagnosis) and the definition of new, realistic and meaningful goals in life; (c) strategies to reach goals, behaviour evaluation and gaining self-efficacy over symptoms (particularly fatigue); (d) the management of negative emotions and positive, negative or illusory thinking styles related to the illness and (e) effective communication (in personal relationships and with health professionals) and the ability to ask for help (13). Further relaxation took place at the session end and advise given about how to implement the learning between sessions.

Physical therapy sessions comprised:

- Four treatments focusing on musculoskeletal issues. Firstly, a full neurological assessment using Medical Expert Information System Computer Kinesiology (MEIS CK) (22) and based on this examination, the system proposed an individually tailored therapy. This consisted of soft tissue treatment, individual exercise and breathing control. Participants were asked to practise the exercises twice a week.
- Three aerobic training sessions on a bicycle ergometer to improve cardiovascular fitness. The intensity and length of load was set individually based on a spiroergometric assessment on a bicycle ergometer and the neurological assessment. Derived training load corresponded to approximately 60% of individual maximal oxygen uptake. The length of load was gradually increased each session, according to the reaction to load, up to a duration of 20–30 min (21). People were advised to do similar activity twice a week.
- Three Motor Programme Activating Therapy (MPAT) sessions, which uses principles of neuroproprioceptive facilitation and inhibition to activate co-contraction of the whole body during everyday activities (23). Participants were given postural correction in lying, sitting, standing up and walking and somatosensory (manual and verbal) stimuli to facilitate realignment and normal movement and reduce compensation strategies. Participants were shown how to continue these techniques in everyday activities.

For the following 6 months, participants were asked to continue with the skills they had learned and to practise self-guided therapy. They were not to attend any new exercise or therapy sessions or adjust their medications unless medically indicated/essential. There was no contact between the study organisers or therapists and the participants to motivate adherence to self-guided therapy during this time in order to replicate real-life experience.

### 2.4 Outcome measures

The referring neurologists collected basic characteristics [age, sex, Body Mass Index, time from disease onset, disease type, Expanded Disability Status Scale (EDSS), current pharmacological treatment]. An assessor independent of the study intervention undertook the primary and secondary outcome measures before the intervention (pre-assessment), after the intervention (post-assessment) and 6 months after the intervention (follow-up assessment).

*Primary outcomes* were self-report questionnaires: Modified Fatigue Impact Scale (MFIS) (24), The Fatigue Scale for Motor and Cognitive Functions (FSMC) (25), Satisfaction with Life Scale (SWLS) (26), Multiple Sclerosis Acceptance Questionnaire (MSAQ) (27), and Beck Depression Inventory (BDI) (28).

Secondary outcomes were absolute and relative values of spirometry (inspiratory vital capacity, VC) and spiroergometric parameters using a bicycle ergometer [maximal muscle performance (W max), maximal pulmonary ventilation (VE max), maximal respiratory exchange ratio (R), maximal oxygen uptake (VO_2_max/kg) and maximal oxygen pulse (VO_2_max/TF)] (20, 21). Steroid hormones (cortisol, cortisone) and neuroactive steroids levels [dehydroepiandrosterone (DHEA), 7-beta-hydroxydehydroepiandrosterone (7β-OH-DHEA), and 7-keto-dehydroepiandrosterone (7-oxo-DHEA)] were obtained from peripheral blood samples and quantified by the LC–MS/MS (liquid chromatography–tandem mass spectrometry) method (29).

### 2.5 Statistical analysis

The data were analysed using both univariate and multivariate methods. Questionnaire, spiroergometry and endocrine results were used as response variables, characteristics of particular patients were used as explanatory variables. Several Wilcoxon tests, following Bonferroni correction, were used within univariate statistics to compare particular response variables between assessment points 1 and 2, and 2 and 3. Repeated measure analysis of variance was used to reveal progress in outcomes during the study, the time and activity of the participants were used as predictors. Data were transformed to meet the assumptions of ANOVA. All univariate tests and output graphics were performed using R and Statistica 13 (30).

Multivariate methods were performed using CANOCO 5 (31). Two approaches were used on the data: unconstrained principal component analysis (PCA) with supplementary explanatory variables to show the relation between measured characteristics and constrained, redundancy analysis (RDA) was employed to find the best explanatory variables (patient characteristics) showing the differences in measured data.

## 3 Results

**Figure 2:**
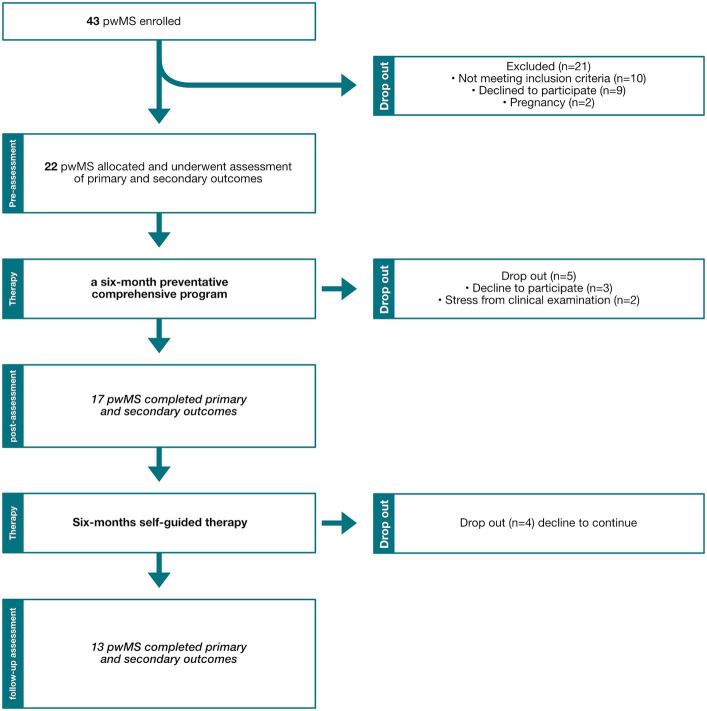
Recruitment flow chart diagram. pwMS, People with multiple sclerosis.

Twenty-two participants were included in the study, 17 completed the 6-month intervention and 13 completed the follow on self-guided therapy (Figure 2). Baseline characteristics are recorded in Table 1.

Complex preventative programme for early MS had the highest impact on the primary outcomes of fatigue and satisfaction with life. Fatigue decreased significantly in both post-assessment (FSMC, *p* = 0.035) and follow-up assessment (FSMC, *p* = 0.007 and MFIS, *p* = 0.035). Satisfaction with life significantly increased (*p* = 0.007) at follow-up.

**Figure 3:**
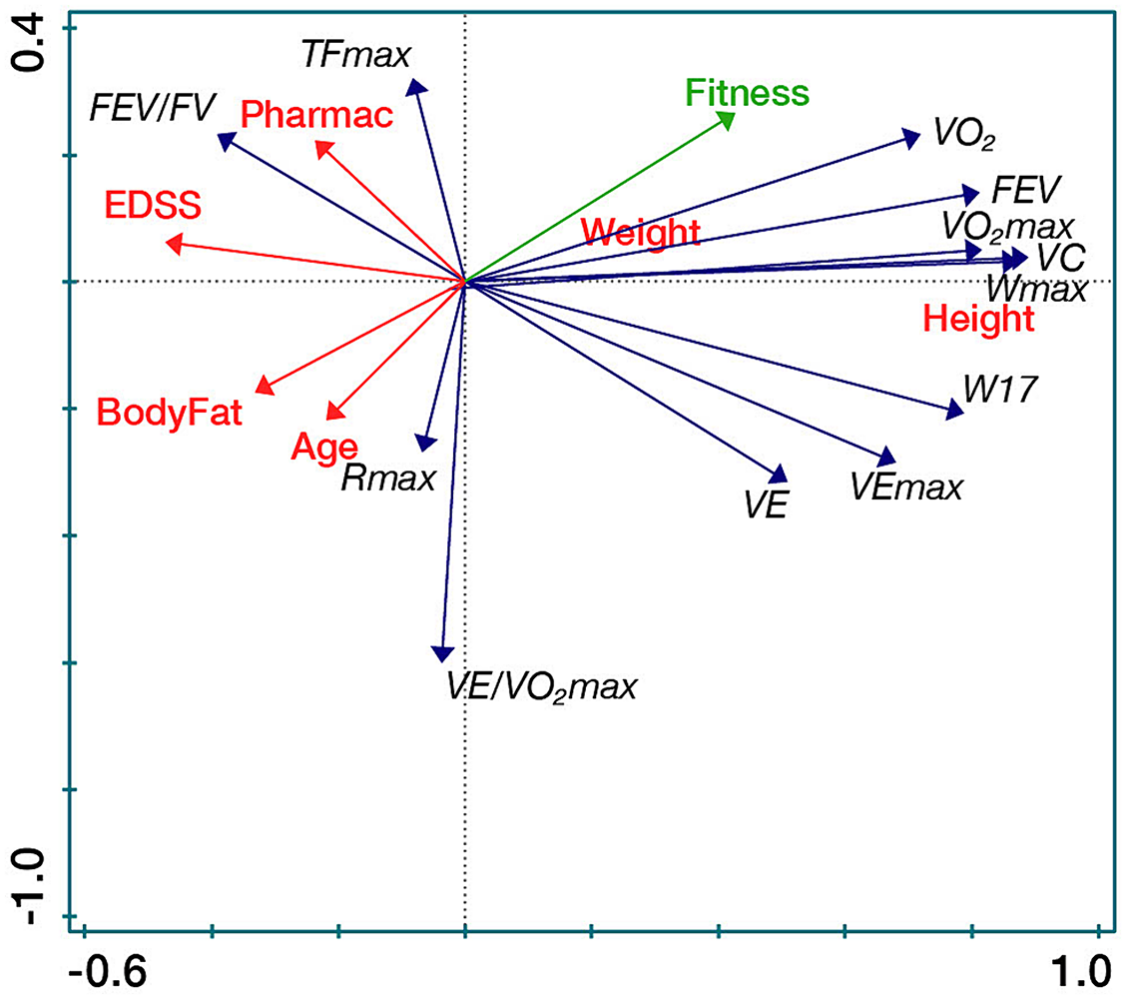
Spirometric and spiroergometric parameters in relation to participants’ characteristics (RDA biplot). VO_2_max/kg [mL/kg/min], Maximal oxygen uptake; VEmax/kg [L/min.kg], Maximal pulmonary ventilation; DHEA [nmol/L], Dehydroepiandrosterone; 7β-OH-DHEA [nmol/L], 7β-Hydroxy-dehydroepiandrosterone; FSMC, Fatigue scale for motor and cognitive functions; and SWLS, Satisfaction with life scale.

The secondary outcomes: spirometry and spiroergometric parameters, vital capacity, maximal oxygen uptake and maximal oxygen pulse were within normal range. Maximal respiratory exchange ratio (93%) and maximal pulmonary ventilation (86%) were slightly reduced compared to normative values. Although the participants had low disability, they had below maximal muscle performance (67%). All parameters stayed at a similar level during the study, but maximal muscle performance reduced (Table 2). Spirometry and spiroergometric parameters improved more in people with lower EDSS (Figure 3). There was a trend to change in steroid hormones. Cortisol decreased in follow-up (median change −38.09 nmol/L) and 7-oxo-DHEA increased in post-assessment (*p* = 0.0638) (Table 2).

**Table 2:** Changes at post intervention and at follow-up.

Questionnaires	M1	M1 → M2	Wilcoxon test	M1 → M3	Wilcoxon test
	Mean	Mean change	*p* value	Mean change	*p* value
SWLS	24 (35)	1	ns	2	0.007
BDI	7 (0)	0	ns	-1	ns
MSAQ	96 (140)	1	ns	-2	ns
MFIS	26 (0)	2	ns	−6	0.035
FSMC	65 (20)	−8	0.035	−9	0.007
Spirometric and spiroergometric parameters	M1	M1 → M2	Wilcoxon test	M1 → M3	Wilcoxon test
	Median (the best value)	Median change	*p* value	Median change	*p* value
VC	4.18 (102%)	−0.05	ns	−0.16	ns
R max	1.04 (93%)	−0.02	ns	0	ns
VO_2_ max/kg	29.4 (100%)	−1.3	ns	−2.2	ns
VO_2_ max/TF	13.3 (123%)	−0.1	ns	−1.6	ns
VE max/Kg	0.99 (86%)	0.14	ns	0.22	ns
Wmax	127.5 (67%)	−2.5	ns	−7.5	ns
Steroids and neuroactive steroids	M1	M1 → M2	Wilcoxon test	M1 → M3	Wilcoxon test
	Median	Median change	*p* value	Median change	*p* value
Cortisol (nmol/L)	499.16	18.12	ns	−38.09	ns
Cortisone (nmol/L)	98.15	14.32	ns	14.84	ns
DHEA (nmol/L)	15.51	0.31	ns	2.64	ns
7β-OH-DHEA (nmol/L)	0.44	0.02	ns	0.09	ns
7-oxo-DHEA (nmol/L)	0.08	0.05	0.0638	0.08	ns

M, Measure; SWLS, Satisfaction with life scale; BDI, Beck depression inventory; MSAQ, Multiple sclerosis acceptance questionnaire; MFIS, Modified fatigue impact scale; FSMC, Fatigue scale for motor and cognitive functions; VC, Vital capacity; Rmax, Maximal respiratory exchange ratio; VO_2_max/kg, Maximal oxygen uptake; VO_2_max/TF, Maximal oxygen pulse; VEmax/kg, Maximal pulmonary ventilation; Wmax, Maximal muscle performance; DHEA, Dehydroepiandrosterone; 7β-OH-DHEA, 7β-hydroxy-dehydroepiandrosterone; and 7-oxo-DHEA, 7-keto-dehydroepiandrosterone.

## 4 Discussion

The main study objective was to evaluate the therapeutic effect of COPREMS. It was shown to reduce fatigue and improve satisfaction with life in newly diagnosed PwMS with improvements sustained at 12 months. Fatigue is a significant problem including in those recently diagnosed with MS (32) and the main reason why pwMS leave the workforce early (33). Therefore, such interventions are important to help people self-manage their condition. The multidimensional approach of this study offered both psychological and physical input which does make it difficult to define which components led to significant improvements, although it was most likely the combination of the cognitive behavioural approach and aerobic training (34). Treatment of musculoskeletal problems were included to reduce pain as there is a positive association between pain and fatigue (35). Participants also received neuroproprioceptive facilitation and inhibition therapy to improve their quality of movement aimed at reducing spasticity and improve walking efficiency, both of which can negatively affect fatigue (21).

Newly diagnosed pwMS are known to experience reduced quality of life (13). In this study, participants did not improve on the Satisfaction with Life Scale at the 6-month post-assessment, but they did show significant improvement at 12-month follow-up. This delayed improvement in quality of life has been previously documented by other authors (19) who used the Multiple Sclerosis Impact Scale and it was suggested that it takes time for people to implement the changes into everyday life. COPREMS led to changes in all self-report outcomes, but perhaps due to the small sample and high variability in all response variables, the differences post-intervention were mostly nonsignificant in the Wilcoxon test results at the follow-up assessments (Table 2). A statistical technique called multiple imputation (36) was applied but the results were similar to the original data analysis and so the original analysis was retained.

Spirometry and spiroergometric parameters did not change in the study. This could be because participants had low disability and relatively good fitness. However, at 12-month follow-up, participants had remained stable and had not deteriorated which might have been expected in a progressive condition such as MS. There was no change in the neuroactive steroid levels. This is in contrast with our previous study which demonstrated a decreasing trend of 7-oxo-DHEA concentration in post-assessment and 7β-OH-DHEA in washout vs. pre-assessment and significant changes occurred in the group who underwent Vojta reflex locomotion (17). This could be because people in the earlier study had higher EDSS scores and reduced levels of neuroactive steroids (Table 3), and therefore had more potential for improvement.

**Table 3:** Level of steroids and neuroactive steroids in newly diagnosed (current study) and in people with moderate MS [previous study ref. (24)].

	Current study	Previous study (24)
Disease duration (years)	2 ± 1.1	12.4 ± 7.4
Mean EDSS	2.41 ± 0.9	4.4 ± 1.5
Cortisol (nmol/L)	499.16	384
Cortisone (nmol/L)	98.15	74.6
DHEA (nmol/L)	15.51	8.58
7β-OH-DHEA (nmol/L)	0.44	0.36
7-oxo-DHEA (nmol/L)	0.08	0.18

Whilst the results show that COPREMS is effective, it was time consuming for participants and for therapists. Of the 33 people meeting the inclusion criteria 9 declined to participate due to the time commitment away from work and other responsibilities. Seventeen of the 22 study participants completed the first 6 months and 13 completed the entire study which further indicates that it is difficult for newly diagnosed people to regularly attend such a therapy programme. Participants were mostly of an age (37.6 ± 10.3) to have both work and family responsibilities as well as trying to maintain their leisure and social activities. Additionally other factors such as fatigue, pain, low mood (1–5), possible medication side-effects and attending other medical appointments all interfere with the ability to participate in such interventions. The Medical Research Council guidance on complex interventions (37) suggests a qualitative study nested to the trial could be considered to assess the impact of such an intervention on both the participants and the health care professionals. This could yield information not only about the elements of behaviour change experienced by the participants but also give useful insights into the experiences of both attending and delivering such a programme.

Despite offering flexible appointment times to maximise attendance, the average attendance rate was 59.6%. Although the sample size was small a further post analysis was undertaken to see if the attendance rate had any impact on outcome. Those who completed at least 60% of the offered therapies were defined as Active participants and those who completed less than 60% were termed Passive. The Active group equated to 10 of 17 participants who completed the 6-month intervention and nine of 13 who completed the follow-on self-guided therapy. The Active group showed greater benefit which concurs with recommendations to be active (11, 38, 39), fatigue decreased significantly at both post-assessment and 12-month follow-up and satisfaction with life improved at post-assessment and reached significance at follow-up. In the Passive group, there was a trend for improvement in these parameters at follow-up (Supplementary Table 1; Supplementary Figures 1, 2). The therapies that were attended most frequently were both elements of physiotherapy (70.6%), aerobic training (64.7%) and group psychotherapy (41.2%). A further consideration could be to offer individual psychotherapy sessions if participants found group sessions difficult however this would lose the benefit of peer support and would demand more resources.

The effectiveness of this intervention both at 6 months and at 12-month follow-up is likely due to the combination of psychological and physical therapy and information giving aimed at reducing fatigue and its impact and improving physical fitness, awareness, and satisfaction with life (a part of quality of life).

Despite the limitations stated, the findings of this study have several important implications for future practise for people newly diagnosed with MS. They highlight the importance of a comprehensive approach early in the disease course and to provide strategies to help people to self-manage their activity levels and make active lifestyle choices.

## 5 Conclusion

This complex preventative programme for newly diagnosed people with multiple sclerosis reduces fatigue and improves satisfaction with life with long-term benefit (at 12-month follow up). People who participated less frequently experienced less benefit.

## Data availability statement

The raw data supporting the conclusions of this article will be made available by the authors, without undue reservation.

## Ethics statement

The studies involving humans were approved by Kralovske Vinohrady University Hospital Ethics Committee, Prague (EK-VP/25/0/2014). The studies were conducted in accordance with the local legislation and institutional requirements. The participants provided their written informed consent to participate in this study.

## Author contributions

NH: Writing – review & editing, Writing – original draft. KB: Writing – review & editing, Writing – original draft. AD: Writing – review & editing, Writing – original draft. TŠ: Writing – review & editing, Writing – original draft. MB: Writing – review & editing, Writing – original draft. LK: Writing – review & editing, Writing – original draft. IŠ: Writing – review & editing, Writing – original draft. ĽB: Writing – review & editing, Writing – original draft. AJ: Writing – review & editing, Writing – original draft. GA: Writing – review & editing, Writing – original draft. KŘ: Writing – review & editing, Writing – original draft.

## In memoriam

Petr Brandejský, Institute of Sports Medicine, First Faculty of Medicine, Charles University who participated in the spirometric and spiroergometric data collection.
