# Supplementary figures and images for: People with newly diagnosed multiple sclerosis benefit from a complex preventative intervention—a single group prospective study with follow up
Source: Front Neurol. 2024 Apr 10;15:1373401. doi: 10.3389/fneur.2024.1373401 (PMC11039797; doi:10.3389/fneur.2024.1373401)

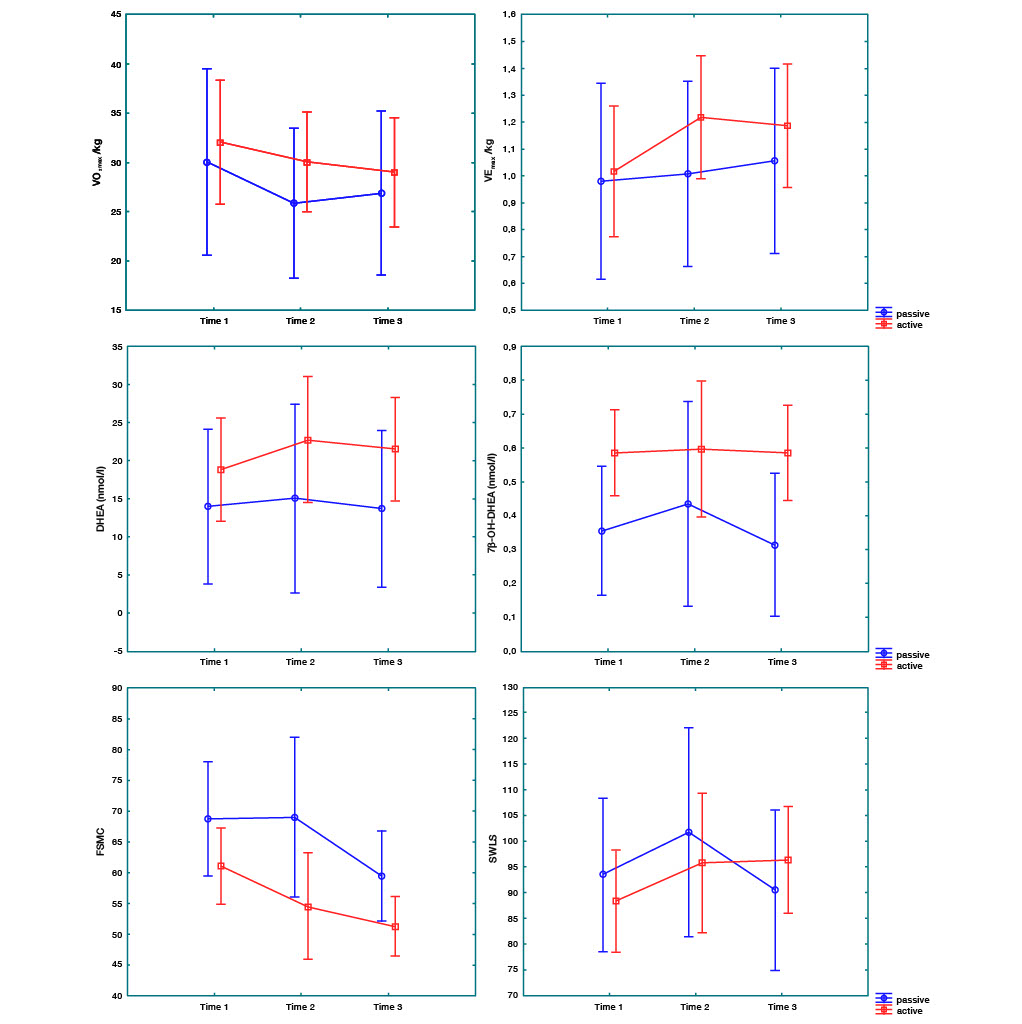

Supplement: Supplementary file 1 [file Image_1.jpeg]

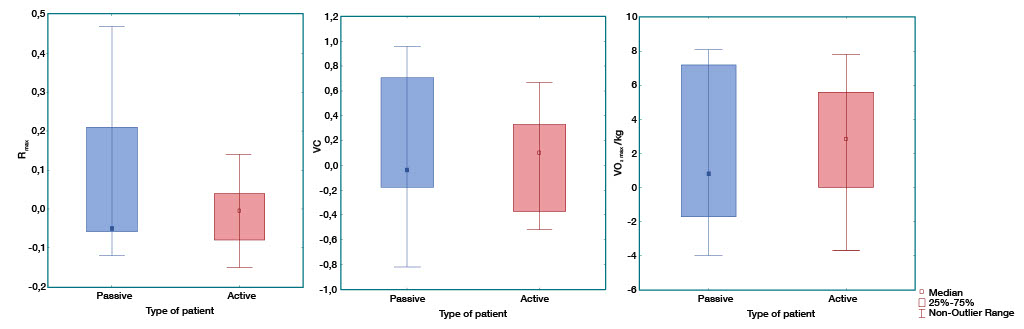

Supplement: Supplementary file 2 [file Image_2.jpeg]
