# Supplementary material for: People with newly diagnosed multiple sclerosis benefit from a complex preventative intervention—a single group prospective study with follow up
Source: Front Neurol. 2024 Apr 10;15:1373401. doi: 10.3389/fneur.2024.1373401 (PMC11039797; doi:10.3389/fneur.2024.1373401)
Supplement: Supplementary file 3 [file Table_1.docx]

**Supplement Table I** *Difference between Active and Passive participants*

|  | Active group | | | | | Passive group | | | | |
| --- | --- | --- | --- | --- | --- | --- | --- | --- | --- | --- |
| **questionnaires** | M1 | M1 → M2 | **Wilcoxon test** | M1 → M3 | Wilcoxon test | M1 | M1 → M2 | **Wilcoxon test** | M1 → M3 | Wilcoxon test |
|  | mean | mean change | p-value | mean change | p-value | mean | mean change | p-value | mean change | p-value |
| SWLS | 23.5 (35)* | 1.5 | ns | 2 | 0.042 | 24 (35)* | -1 | ns | 1.5 | 0.097 |
| BDI | 7 (0)* | 0 | ns | 0 | ns | 8 (0)* | -2 | ns | -3.5 | ns |
| MSAQ | 92.5 (140)* | 4.5 | ns | 1.5 | ns | 99 (140)* | -5 | ns | -10 | ns |
| MFIS | 27.5 (0)* | 1 | ns | -9.5 | ns | 26 (0)* | 2 | ns | -2.5 | ns |
| FSMC | 63 (20)* | -7 | 0.021 | -13 | 0.044 | 71 (20)* | -8 | ns | -10 | 0.097 |
| **Spirometer and spiroergometric parameters** | **M1** | **M1 → M2** | **Wilcoxon test** | M1 → M3 | Wilcoxon test | **M1** | **M1 → M2** | **Wilcoxon test** | M1 → M3 | Wilcoxon test |
|  | **median** | **median change** | **p-value** | median change | p-value | **median** | **median change** | **p-value** | median change | p-value |
| VC | 3.95 (101 %) | 0.01 | ns | -0.2 | ns | 4.39 (102 %) | -0.03 | ns | 0.22 | ns |
| Rmax | 1.04 (93.5 %) | -0.03 | ns | 0.07 | ns | 1.05 (93 %) | -0.02 | ns | -0.02 | ns |
| VO_2_max/kg | 30.80 (106.5 %) | -3.75 | ns | -3.2 | ns | 28.9 (89 %) | 0 | ns | 1.95 | 0.07 |
| VO_2_max/TF | 12.85 (119 %) | -0.3 | ns | -1.55 | ns | 14.5 (124 %) | -0.4 | ns | 1.05 | ns |
| VEmax/kg | 1.15 (96.5 %) | 0.09 | ns | 0.27 | ns | 0.98 (75 %) | 0.03 | ns | -0.04 | ns |
| Wmax | 135 (72 %) | -2.5 | ns | -15 | ns | 112.5 (67 %) | -7.5 | ns | -5 | ns |
| **Steroids and neuroactive steroids** | **M1** | **M1 → M2** | **Wilcoxon test** | M1 → M3 | Wilcoxon test | **M1** | **M1 → M2** | **Wilcoxon test** | M1 → M3 | Wilcoxon test |
|  | **median** | **median change** | **p-value** | median change | p-value | **median** | **median change** | **p-value** | median change | p-value |
| Cortisol (nmol/l) | 488.2 | 84.69 | ns | 64.88 | ns | 499.16 | 7.62 | ns | -38.09 | ns |
| Cortisone (nmol/l) | 100.01 | 1.17 | ns | 18.4 | ns | 98.15 | 14.57 | ns | 10.67 | ns |
| DHEA (nmol/l) | 17.44 | -0.31 | 0.0644 | 4.08 | ns | 13.97 | 1.85 | ns | 1.12 | ns |
| 7β-OH-DHEA (nmol/l) | 0.52 | 0 | ns | 0.02 | ns | 0.36 | -0.03 | ns | -0.05 | ns |
| 7-oxo-DHEA (nmol/l) | 0.07 | 0.08 | ns | 0.1 | ns | 0.1 | 0.03 | ns | -0.03 | ns |
